# The Muscularity-Oriented Eating Test, Drive for Muscularity Scale, and Muscle Dysmorphic Disorder Inventory among Chinese Men: Confirmatory Factor Analyses

**DOI:** 10.3390/ijerph182111690

**Published:** 2021-11-07

**Authors:** Jinbo He, Stuart Murray, Emilio J. Compte, Jianwen Song, Jason M. Nagata

**Affiliations:** 1 School of Humanities and Social Science, The Chinese University of Hong Kong, Shenzhen 518172, China; jianwensong@link.cuhk.edu.cn; 2 Department of Psychiatry and Behavioral Sciences, Keck School of Medicine, University of Southern California, Los Angeles, CA 90033, USA; stuartmu@usc.edu; 3 Eating Behavior Research Center, School of Psychology, Universidad Adolfo Ibáñez, Santiago 7941169, Chile; ejcompte@gmail.com; 4 Research Department, Comenzar de Nuevo Treatment Center, Monterrey 66220, Mexico; 5 Division of Adolescent & Young Adult Medicine, Department of Pediatrics, University of California, San Francisco, CA 94158, USA; jason.nagata@ucsf.edu

**Keywords:** disordered eating, muscularity, validation, psychometric properties, Chinese

## Abstract

Research on eating disorders (EDs) and body image disturbances has focused mostly on females from Western countries, and little is known about EDs in male populations in China, which is partially due to the lack of validated assessment measures. The current work aims to translate the Muscularity-Oriented Eating Test (MOET), Drive for Muscularity Scale (DMS) and Muscle Dysmorphic Disorder Inventory (MDDI) into Chinese and examine their psychometric properties. The factor structures, reliability and validity of the translated scales were examined with two samples: male university students (*n* = 295, *M_ag_*_e_ = 18.92 years) and general adult men (*n* = 406, *M_age_* = 28.53 years). With confirmatory factor analyses, the original factor structures are replicated for the MOET, DMS and MDDI. The results also support the adequate internal consistency for both samples. Strong evidence of convergent and incremental validity for the three measures is also found in both samples. Overall, the three measures prove to be good instruments for use among Chinese male university students and general adult men.

## 1. Introduction

Eating disorders (EDs) are severe mental illnesses characterized by disturbances in eating behaviors and body image [1]. Traditionally, research on EDs and their key risk factors—body image disturbances—was mainly conducted among women [2,3,4]. However, there has been ample evidence showing that EDs and body image disturbances increasingly occur in men, and the presentations of EDs and body image disturbances among men are often distinct to those observed in women [5]. Specifically, according to the previous literature [3,5,6], EDs and body image disturbances among women are typically thinness-oriented (i.e., drive for thinness), while EDs and body image disturbances among men are typically muscularity-oriented (i.e., drive for muscularity).

The majority of the existing measures that are widely used for assessing ED symptomatology and body image disturbances are thinness-focused, such as the Eating Attitude Test [7], the Eating Disorder Examination [8] and the Eating Disorder Inventory [9]. Thus, the measures are not sensitive enough to capture the core features of disordered eating or body image disturbances among men [5].

To fill this gap, Murray et al. [6] developed and validated the Muscularity-Oriented Eating Test (MOET) with a sample of 511 undergraduate men in the US. Specifically, the MOET contains 15 items that are rated on a 5-point Likert-type scale from 0 (“never true”) to 4 (“always true”). In the work of Murray et al. [6], the MOET showed a unidimensional structure, high internal consistency (omega = 0.92~0.93), high test-retest correlation (*r* = 0.75) and construct validity, as indicated by large correlations between the MOET scores and theoretically closely related measures (e.g., drive for muscularity) but small correlations between the MOET scores and theoretically weak or unrelated measures (e.g., socially desirable response). Currently, the MOET has been translated into Spanish, and the Spanish version replicated the unidimensional structure and showed good psychometric properties in a sample of Argentinian college men [10].

To date, research on EDs or body image disturbances among men is mainly conducted in Western societies among adolescents or young adults of Caucasian ethnicity [2]. However, there has been a rising trend of ED prevalence in Asian countries, including China [11,12], and more attention has been called to EDs in Chinese men [13]. Thus, it is imperative and significant to conduct more research on EDs in Chinese male populations for exploring whether there are unique clinical manifestations, risk factors and health consequences of EDs in Chinese men.

Furthermore, as body image is a key contributing factor of EDs [14], it is also important to assess male-specific body image disturbances to explore the etiology of EDs among men. However, different from women and girls, the major body image concern among men and boys is drive for muscularity [15,16,17], which refers to an individual’s desire to achieve an ideal muscular body [15]. Previous studies have shown that drive for muscularity is a significant risk factor for EDs among men [3,18,19]. Furthermore, drive for muscularity is also a significant risk factor or characteristic of muscle dysmorphia (MD) [20,21,22]. Specifically, as a subtype of body dysmorphic disorder, muscle dysmorphia (MD) represents the pathological extreme pursuit of muscularity and is characterized by a pervasive belief or fear around insufficient muscularity [23,24]. Muscle dysmorphia is mostly researched among men, and it leads to considerable functional impairment [25].

However, to the best our knowledge, there have been no such measures available in Chinese for assessing male-specific body image disturbances or muscle dysmorphia. To bridge this gap, another two widely used measures assessing male-specific body image disturbances were also chosen to be translated and validated in the current study: the Drive for Muscularity Scale (DMS) [15] and the Muscle Dysmorphic Disorder Inventory (MDDI) [26].

Specifically, the DMS is a 15-item self-report questionnaire rated on a 6-point Likert-type scale with responses from 1 (“always”) to 6 (“never”), which has two subscales: the muscle-oriented body image subscale (MBI, or the muscularity attitudes subscale; 7 items) and the muscle-oriented behavior subscale (MB, or the muscularity behavior subscale; 8 items) [15]. All items are reverse-coded, in which the total score (or mean score) of the 15 items then represents an individual’s overall drive for muscularity. The higher the total score, the stronger the attitudes and behaviors of drive for muscularity. Currently, the DMS has been translated into a number of different languages and shown adequate reliability and validity, including Romanian [27], Spanish [28,29], French [30], Turkish [31], Portuguese [32] and Japanese [33].

The MDDI [26] contains 13 items that comprise three subscales: drive for size (DFS, 5 items), appearance intolerance (AI, 4 items) and functional impairment (FI, 4 items). The MDDI is rated on a 5-point Likert-type scale with responses from 1 (“never”) to 5 (“always”). A total score can be derived by the sum of all items or all subscales. Higher total scores indicate more symptoms of muscle dysmorphia. The MDDI has also been translated into a number of different languages and demonstrated good psychometric properties in samples from different countries, including Portuguese [34], Italian [35], German [25], Turkish [36] and Spanish [37].

Overall, the current study aimed to translate and validate three measures (i.e., the MOET, DMS and MDDI) to facilitate future research on EDs and body image disturbances among Chinese men. We hypothesized that (1) the MOET would have a unidimensional factor structure, the DMS would have a two-factor structure, and the MDDI would have a three-factor structure; (2) the three measures would have adequate reliably as indicated by internal consistency; (3) the total scores of the three measures would be closely related to each other; and (4) the total scores of the three measures would be positively related to traditional eating disorder symptomology, psychological impairment, psychological distress and thinness-oriented body dissatisfaction.

## 2. Materials and Methods

### 2.1. Participants and Procedure

The protocol of data collection was approved by the Institutional Review Board of the Chinese University of Hong Kong, Shenzhen. We used two male samples for the current study: university students and male adults from the general population.

#### 2.1.1. University Students

For the sample of male university students, the survey was conducted in a paper-and-pencil format in a comprehensive university in Hunan province. Psychological teachers introduced the information about the project (i.e., the study design) to 1223 first- and second-year undergraduate students during class time and invited them to participate. Two attention check questions were used to ensure the quality of the survey. Finally, 1059 students met our inclusion criterion (i.e., ≥18 years old) and provided written informed consent. Of those, 812 (36.3% males; 295) passed the two attention check questions and were included in the study. As the current study is about male university students’ body image and eating behaviors, only the sample of 295 males was used in the current study, and they had a mean (SD) age of 18.92 (1.04) years (range: 18~23 years). The mean (SD) of body mass index (BMI) based on self-reported height and weight [38,39] was 22.13 (3.92) kg/m^2^ (range: 15.57~36.73 kg/m^2^).

#### 2.1.2. Male Adults from the General Population

To recruit general male adults, we used an online Chinese survey platform, Credamo, which is similar to Qualtrics Online Sample. Credamo recruits participants from all over China. In the online survey, participants provided informed consent by selecting the “agree to participate” option at the end of the first page. If any participant did not give his informed consent, the survey would end immediately. Moreover, the participants were also informed that they could stop at any time during the survey by closing the survey page. Two attention check questions were used to ensure the quality of the survey, and surveys from the participants who failed to pass the two attention check questions were removed automatically by Credamo. By setting the criteria of the target sample (i.e., ≥18 years old), 532 male adults filled out our survey, and 406 of them were included by passing the two attention check questions. The included participants’ ages ranged from 18 to 53 years old, with a mean (SD) of 28.53 (5.56) years. Their BMIs ranged from 15.87 to 32.96 kg/m^2^, with a mean (SD) of 22.28 (2.80) kg/m^2^.

According to the guidelines to estimate the sample size for confirmatory factor analysis [40], for a power of 0.80, a root mean square error of approximation value of 0.05 and an alpha level of 0.05, a minimum sample size of 174 participants was required for the MOET, while it was 175 for the DMS and 215 for the MDDI. Thus, the sample sizes of 295 and 406 were adequate for the purposes of the current work.

### 2.2. Translation

The Chinese versions of the MOET, DMS and MDDI were obtained based on the procedures recommended in the previous literature for cross-cultural translation of scales [41]. Specifically, the English versions of the three measures were first translated to Chinese (Mandarin) by a Ph.D. student and a master’s student independently, and both students were proficient in English. Then, the two translations for each of the three measures were compared, and the differences were discussed within the research team, leading to a synthesized translation for each of the three measures. Back-translation was conducted independently by two new bilingual master’s students who had no prior knowledge of the original English measures. Afterward, the forward- and back-translations were evaluated by the research team. The research team, as an expert review committee, consisted of the four translators and three experts in eating behaviors and body image. In addition, we successfully involved the developers of the MOET and MDDI into the translation process by requesting them to evaluate the equivalence of the back-translations and the original English versions. As the research team did not identify translation issues, no modifications to the translations were made. Next, the preliminary Chinese translations were administered to 30 male university students (*M_age_* = 19.80 years, SD = 1.03) for evaluating the understandability of the items. They were asked to evaluate the understandability of the items on a 5-point Likert scale from 1 (“I do not understand at all”) to 5 (“I completely understand, and I have no doubts”). The results showed that the understandability of the MOET ranged from 4.03 to 4.77, the understandability of the MDDI ranged from 3.50 to 4.53, and the understandability of the DMS ranged from 4.20 to 4.67. Based on Campos et al. [42], means of understandability ≥3 were considered adequate understandability, representing degrees of understanding of approximately 80%. As such, the preliminary Chinese translations (i.e., the C-MOET, C-DMS and C-MDDI) were used in the subsequent studies. 

### 2.3. Measures

In addition to the three measures (i.e., the MOTE, DMS and MDDI) being validated in the current study, the following measures were used for examining the construct validity of the three measures.

#### 2.3.1. Psychosocial Impairment

The Clinical Impairment Assessment Questionnaire (CIA 3.0) [43] was used to assess psychosocial impairment secondary to eating disorder symptomatology. The CIA 3.0 contains 16 items which are rated on a 4-point Likert-type scale from 0 (“not at all”) to 3 (“always”). The summation of the 16 items is used as the overall indicator of psychological impairment, with higher total scores indicating greater psychological impairment. Previous studies showed that the CIA 3.0 had good psychometric properties [44]. In the current study, the Chinese version of the CIA 3.0 (C-CIA 3.0) was used. The CIA 3.0 was translated into Chinese with similar procedures to those described in the translation section, and the only difference was that the preliminary C-CIA 3.0 was administrated to adolescent boys, adolescent girls, undergraduate men and undergraduate women for evaluating the meanings, clarity and understandability of the items. Cronbach’s α of the CIA 3.0 was 0.94 and 0.90 for the samples of university students and general adults, respectively.

#### 2.3.2. Psychological Distress

The 6-item Kessler Psychological Distress Scale (K6) [45] was used to assess psychological distress during the past month. The items are rated on a 5-point Likert scale from 1 (“none of the time”) to 5 (“all of the time”). Higher total scores indicate greater psychological distress. The Chinese version of the K6 was used in the current study, and it had shown good psychometric properties in Chinese populations [46]. In the sample of university students, Cronbach’s α of the K6 was 0.88, and in the sample of general adults, Cronbach’s α of the K6 was 0.82.

#### 2.3.3. Thinness-Oriented Eating Disorder Symptomatology

The 12-item self-reporting Eating Disorder Examination Questionnaire (EDE-QS) [47] was used to examine traditional eating disorder symptomatology (i.e., thinness-oriented disordered eating). The response scale ranges from 0 to 3. The summed scores of the 12 items are the total score. Higher total scores indicate a higher level of traditional eating disorder symptomatology. Recent findings suggest that the Chinese version of the EDE-QS showed strong reliability and validity [48]. In the current study, the Cronbach’s α of the EDE-QS was 0.92 and 0.88 for the samples of university students and general adults, respectively.

#### 2.3.4. Thinness-Oriented Body Dissatisfaction

The 9-item body dissatisfaction subscale of the Eating Disorder Inventory (EDI-BD) [49] was used to measure thinness-oriented body dissatisfaction. The items are rated on a 6-point Likert-type scale from 1 (“never”) to 6 (“always”). Higher total scores represent a higher level of body dissatisfaction. Adequate reliability and validity of the EDI-1 were reported for Chinese male university students [50]. Cronbach’s α of the EDI-BD was 0.90 and 0.92 and for the university sample and the general adult sample, respectively.

### 2.4. Statistical Analysis

All data analyses in the current work were carried out via the R 4.0.0 with the lavaan package version 0.6–7 [51] and psych package version 2.0.9 [52]. Specifically, confirmatory factor analysis (CFA) was used to confirm the one-factor structure of the MOET [6], two-factor structure of the DMS [15] and three-factor structure of the MDDI [26]. To estimate the CFA models, the diagonally weighted least squares (WLSMV) estimator was adopted, as it was recommended for Likert-type rating scales [53]. To evaluate the model fit of CFA, according to Hu and Bentler [54] and Shi et al. [55], the following fit indicators were reported: the comparative fit index (CFI; close to or >0.95 indicates a good fit, but 0.90 indicates an acceptable fit), Tucker–Lewis index (TLI; close to or >0.95 indicates a good fit, but 0.90 indicates an acceptable fit) and the standardized root mean square residual (SRMR; close to or <0.06 indicates a good fit, but 0.10 indicates a mediocre fit). It should be noted that we decided not to report the root mean square error of approximation (RMSEA) because it is unreliable and inferior to SRMR when used in CFA with ordinal responses (e.g., Likert-type responses) [55]. We also used the multi-group CFA method to assess measurement invariance by sample type (university students vs. general adults). According to Cheung and Rensvold [56], CFI < 0.01 and SRMR < 0.030 indicate invariance between two nested models (e.g., configural model vs. metric model and metric model vs. scalar model).

To examine the reliability, we calculated the McDonald’s omega values for the MOET, DMS subscales and MDDI subscales [57]. Furthermore, we evaluated the concurrent validity of the MOET, DMS and MDDI by exploring the correlations between the scores of the three scales being validated and the theoretically correlated measures. Based on previous research (e.g., [6,15,26,27,34,37]), we expected significant and positive associations among the MOET scores, DMS scores, MDDI scores, CIA scores, K6 scores, EDE-QS scores and EDI-BD scores. According to Cohen [58], correlations of 0.1, 0.3, and 0.5 were considered small, medium and large, respectively.

Finally, incremental validity was explored with hierarchical multiple regression analyses to determine whether the scores of the three measures being validated explained the unique variances in the key criterion variables after controlling for confounding variables. Specifically, since both muscularity-oriented disordered eating and muscle dysmorphic disorder symptoms, as measured by the MOET and MDDI, respectively, were already “outcome or criterion” variables, we cared about whether the MOET scores and the MDDI scores could uniquely explain the variances in psychosocial impairment and psychological distress when the confounding variables (e.g., age, BMI, traditional eating disorder symptomatology and traditional thinness-oriented body dissatisfaction) were controlled. Regarding the DMS scores, the criterion variables selected were muscularity-oriented disordered eating and muscle dysmorphic disorder symptoms, and the cofounding variables were age, BMI, traditional eating disorder symptomatology and traditional thinness-oriented body dissatisfaction.

## 3. Results

### 3.1. Confirmatory Factor Analysis and Internal Consistency

#### 3.1.1. Muscularity-Oriented Eating Test

For the university sample, the results of CFA showed that the one-factor structure had an adequate model fit, with χ^2^ = 281.68 (*df* = 90, *p* < 0.001), CFI = 0.98, TLI = 0.98 and SRMR = 0.05. For the general adult sample, the one-factor structure also had an adequate model fit, with χ^2^ = 345.74 (*df* = 90, *p* < 0.001), CFI = 0.95, TLI = 0.94 and SRMR = 0.06. The standardized factor loadings are shown in Table 1. The internal consistency reliability, as indicated by McDonald’s omega, was 0.92 with a 95% CI [0.89, 0.93] and 0.90 with a 95% CI [0.88, 0.91] for the university sample and the general adult sample, respectively.

#### 3.1.2. Drive for Muscularity Scale

Within the university sample, the two-factor structure had an acceptable model fit, with χ^2^ = 502.08 (*df* = 89, *p* < 0.001), CFI = 0.96, TLI = 0.95 and SRMR = 0.11. As for the general adult sample, the two-factor structure had a marginally acceptable model fit, with χ^2^ = 714.71 (*df* = 89, *p* < 0.001), CFI = 0.91, TLI = 0.89 and SRMR = 0.09. Since certain items of the DMS share similar wording [27], the modification indices (M.I.) were checked. Based on the M.I. values, the covariances among three items (i.e., Items 13, 14, and 15) were freely estimated one by one, as these items had similar wording (i.e., I think that my arms (for Item 13), chest (for Item 14) or legs (for Item 15) are not muscular enough). The modified model showed an adequate model fit, with χ^2^ = 493.63 (*df* = 86, *p* < 0.001), CFI = 0.94, TLI = 0.93 and SRMR = 0.08. The standardized factor loadings are described in Table 2. For the university sample, the McDonald’s omega values were 0.93 with a 95% CI [0.91, 0.94] and 0.93 with a 95% CI [0.90, 0.95] for the muscle-oriented body image subscale and the muscle-oriented behavior subscale, respectively. For the general adult sample, the McDonald’s omega values were 0.84 with a 95% CI [0.81, 0.87] and 0.87 with a 95% CI [0.84, 0.88] for the muscle-oriented body image subscale and the muscle-oriented behavior subscale, respectively.

#### 3.1.3. Muscle Dysmorphic Disorder Inventory

In both the university sample and general adult sample, the three-factor structure of the MDDI had an acceptable model fit, with χ^2^ = 317.95 (*df* = 62, *p* < 0.001), CFI = 0.95, TLI = 0.94 and SRMR = 0.09 for the university sample and χ^2^ = 271.13 (*df* = 62, *p* < 0.001), CFI = 0.94, TLI = 0.92 and SRMR = 0.07 for the general adult sample. The standardized factor loadings of the model were described in Table 3. For the university sample, the McDonald’s omega values were 0.80 with a 95% CI [0.80, 0.84], 0.81 with a 95% CI [0.77, 0.85] and 0.87 with a 95% CI [0.83, 0.90] for the drive for size subscale, the appearance intolerance subscale and the functional impairment subscale, respectively. For the general adult sample, the McDonald’s omega values were 0.81 with a 95% CI [0.78, 0.84], 0.69 with a 95% CI [0.64, 0.74] and 0.83 with a 95% CI [0.80, 0.86] for the drive for size subscale, the appearance intolerance subscale and the functional impairment subscale, respectively.

### 3.2. Invariance and Mean Difference Tests

#### 3.2.1. Muscularity-Oriented Eating Test

To use the WLSMV estimator for the invariance tests, the missing patterns of the response categories should be the same across different samples. However, there was no response category missing in the general adult sample, but there was a missing response category of 4 (“always true”) in Item 8 (“I have pre-cooked several meals in advance to ensure that I do not deviate from my diet plan.”) for the university sample. Thus, the invariance and mean difference tests were not conducted for the MOET.

#### 3.2.2. Drive for Muscularity Scale

As shown in Table 4, all indices suggested that configural, metric and scalar invariance were supported across the samples for the DMS. Latent mean difference tests showed that the general adults had significantly higher latent means for MBI (Cohen’s *d* = 1.02; *p* < 0.001) and MB (Cohen’s *d* = 1.32; *p* < 0.001) than those of the university students.

#### 3.2.3. Muscle Dysmorphic Disorder Inventory

The results in Table 4 show that configural, metric and scalar invariance were also supported across the samples for the MDDI. Latent mean difference tests showed that the general adults had significantly higher latent means of DS (Cohen’s *d* = 1.47; *p* < 0.001), AI (Cohen’s *d* = 0.69; *p* < 0.001) and FI (Cohen’s *d* = 1.37; *p* < 0.001) than those of the university students.

### 3.3. Concurrent Validity

#### 3.3.1. Muscularity-Oriented Eating Test

As shown in Table 5, for the university sample, there were medium to large positive correlations with the theoretically closely related constructs, namely the drive for muscularity, muscle dysmorphic disorder symptoms, psychological impairment and traditional eating disorder symptomatology. For the general adult sample, the MOET scores had large positive correlations with all the theoretically closely related constructs. For both samples, the MOET scores also had a medium correlation with psychological distress and a small correlation with thinness-oriented body dissatisfaction, but the MOET scores were not significantly related to BMI or age.

#### 3.3.2. Drive for Muscularity Scale

As shown in Table 5, for both samples, the DMS scores had significant and large or close-to-large correlations with muscle-oriented disordered eating, muscle dysmorphic disorder symptoms, psychological impairment and traditional eating disorder symptomatology. Moreover, for both samples, the DMS scores had a small but significant positive correlation with thinness-oriented body dissatisfaction, and the DMS scores were not significantly related to BMI.

#### 3.3.3. Muscle Dysmorphic Disorder Inventory

As seen in Table 5, for both samples, the MDDI scores showed significant and large or close-to-large correlations with muscle-oriented disordered eating, the drive for muscularity, psychological impairment, traditional eating disorder symptomatology and psychological distress. In addition, for both samples, the MDDI scores were not significantly related to BMI or age.

### 3.4. Incremental Validity

Table 6 presents the hierarchical regression models with the MOET, DMS and MDDI scores entered in the second step. Specifically, for both samples, the inclusion of the MOET and MDDI scores significantly increased the variance explained in psychological impairment, and the inclusion of the DMS scores significantly increased the variance explained in muscularity-oriented disordered eating and muscle dysmorphic disorder symptoms.

## 4. Discussion

The need to further study male EDs and body image disturbances among Chinese men has been made clear [13]. However, due to the lack of validated instruments assessing eating and body image disturbances among Chinese men, limited research about these topics is possible at this time in China. Thus, the current study aimed to translate three measures (i.e., the MOET, DMS and MDDI) that specifically assess eating and body image disturbances among men and examine the psychometric properties of the Chinese translations of the three measures. The results showed that all measures presented good psychometric properties with both a sample of male university students and a sample of general male adults.

Specifically, in line with our hypotheses, the respective one-factor structure [6], two-factor structure [15] and three-factor structure [26] of the MOET, DMS and MDDI were successfully confirmed via CFA in the current study. This indicates that the structures of the scales are stable across different cultures, especially considering that the factor structures of the DMS and MDDI have been successfully replicated in a number of samples from different countries. Thus, future researchers may consider conducting cross-cultural measurement invariance tests (e.g., [59]) for the three measures to explore whether these measures can be used for cross-cultural comparisons in disordered eating and body image disturbances among men, which can greatly forward our understanding of the cultural differences in these areas.

The McDonald’s omega values for the MOET, DMS subscales and MDDI subscales were acceptable in the current two samples, suggesting that the scores of the three measures had adequate internal consistency reliability, which is in line with previous validation studies (e.g., [6,15,26]). In the present work, as hypothesized, the scores of the three measures were found to be significantly and positively related to each other. These large inter-correlational findings provide evidence for the convergent validity of the three measures, since all three constructs being measured (i.e., muscularity-oriented disordered eating, drive for muscularity and muscle dysmorphic disorder symptoms) are focused on muscularity.

We also found that the total scores of the three measures were closely related to traditional eating disorder symptomology. These findings are also consistent with the previous literature [6,10]. As muscularity concerns in men include not only muscularity but also leanness [5], it is not surprising to find large correlations between the scores of the three measures and traditional eating disorder symptomology, which is thinness-oriented. Furthermore, the close relationships between the scores of the three measures and psychosocial impairment secondary to eating disorder symptomology suggest the potential negative effects from muscularity-oriented disordered eating and body image disturbances on men’s quality of life. Together, with the medium correlations between the total scores of the three measures and psychological distress, these correlational findings warrant interventions for EDs and body image disturbances among Chinese men. 

The results also showed that thinness-oriented body dissatisfaction, as measured by the EDI-BD, had small to medium associations with the scores of the three measures. These findings are not surprising, since the EDI-BD focuses on body weight and shape dissatisfaction due to body fat [49], while the three measures focus on muscularity. Moreover, the scores of the three measures contributed a significant amount of unique variance to each criterion variable, indicating the good incremental validity of the three measures.

Finally, we found that the subscale scores of the DMS and MDDI were significantly higher in general adult men than university students, indicating that Chinese general adult men may have more muscularity-oriented body image disturbances. Except for the sampling differences in recruiting the two samples (paper and pencil in university students vs. online in general adults), the large differences in professional status may also help explain the score differences in the DMS and MDDI. Specifically, different from Chinese university students who generally live in university dormitories and rely on the monthly financial support from their parents to live (called “monthly living fees”) [60], the majority of the general adults in the current study were employed and had stable income. Thus, general adults should have more time and money to get involved in activities to strengthen their muscles.

The current work is not free from limitations. First, because the sample sizes were only enough for us to run CFAs, we did not conduct exploratory factor analysis by splitting the samples as recommended in validating body image related measures [61]. Thus, the current study only confirmed the original factor structures shown in the previous literature, and future studies with larger sample sizes are needed to explore whether there are different factor structures in the three measures in the Chinese context. However, it should also be noted that as the original factor structures of the DMS and MDDI had been replicated in a wide variety of populations, and a recent validation of MOET also replicated the original single-factor structure [10], we chose to run CFAs only for confirming the original factor structures. Second, our study was limited to Chinese adult men. Thus, the findings cannot be generalized to Chinese women, adolescent boys or clinical populations (i.e., patients with eating disorders or muscle dysmorphia). Third, the invariance tests by sample type were not conducted, as certain important factors, such as sexual orientation [62], were not included because we did not collect such information. However, future studies should be conducted to check whether these measures can be used to make group comparisons. Fourth, the test-retest reliability of the measures was not assessed. Thus, the test-retest reliability of the three measures remains unknown. Finally, as a common issue in survey research, socially desirable responding [63] might have affected the scores obtained in the scales, so future studies may test whether socially desirable responding is an important issue by including an instrument to assess socially desirable response tendencies (e.g., [64]). Future researchers are highly encouraged to conduct research by considering the limitations of the current work.

## 5. Conclusions

In summary, the three measures (i.e., the MOET, DMS and MDDI) showed good psychometric properties in two samples of Chinese adult men. To date, there has been limited research related to muscularity-oriented disordered eating and body image disturbances in China, and the translation and validation of the three measures can serve as the first step to further understanding these areas of research within the Chinese context.

## Figures and Tables

**Table 1:** Standardized factor loadings and factor correlation for the Muscularity-Oriented Eating Test.

Items	Standardized Factor Loadings	
	University Sample	General Adult Sample
1. I have recorded the macro-nutritional values of everything that I ate.	0.47	0.55
2. I have used meal replacement supplements when I felt full.	0.52	0.62
3. What I ate has influenced how I think about myself as a person.	0.73	0.70
4. There are definite foods I have avoided eating due to worry about how they might affect my shape or weight.	0.75	0.75
5. I have felt less anxious about eating out if I knew the macro-nutritional content of the food at the restaurant.	0.79	0.70
6. I have taken my own food out with me to social events in case the food on offer is inconsistent with my diet plan.	0.82	0.73
7. I cannot achieve my ideal body unless I exert complete control over everything I eat.	0.66	0.61
8. I have pre-cooked several meals in advance to ensure that I do not deviate from my diet plan.	0.87	0.73
9. I have continued eating despite feeling full in an attempt to influence my muscularity.	0.75	0.46
10. I have felt anxious when I run out of protein-based supplements.	0.91	0.67
11. I have been deliberately trying to limit the overall volume of some foods so that my muscles look more defined.	0.86	0.80
12. If I broke any of my food rules, I attempted to make up for it with my next meal.	0.80	0.67
13. I have felt anxious about others knowing the rules I have around what I eat.	0.89	0.64
14. Other people do not seem to understand how important my food choices are to me.	0.87	0.60
15. Ensuring proper adherence to my dietary ideals is more important to me than adhering to a work schedule.	0.76	0.62

**Table 2:** Standardized factor loadings and factor correlation for the Drive for Muscularity Scale.

Items	Standardized Factor Loadings			
	University Sample		General Adult Sample	
	MBI	MB	MBI	MB
1. I wish that I were more muscular.	0.77		0.82	
7. I think I would feel more confident if I had more muscle mass.	0.89		0.83	
9. I think that I would look better if I gained 10 pounds in bulk.	0.60		0.45	
11. I think that I would feel stronger if I gained a little more muscle mass.	0.88		0.82	
13. I think that my arms are not muscular enough.	0.89		0.61	
14. I think that my chest is not muscular enough.	0.90		0.64	
15. I think that my legs are not muscular enough.	0.88		0.53	
2. I lift weights to build up muscle.		0.76		0.76
3. I use protein or energy supplements.		0.85		0.81
4. I drink weight gain or protein shakes.		0.87		0.79
5. I try to consume as many calories as I can in a day.		0.64		0.67
6. I feel guilty if I miss a weight training session.		0.89		0.79
8. Other people think I work out with weights too often.		0.94		0.73
10. I think about taking anabolic steroids.		0.85		0.66
12. I think that my weight training schedule interferes with other aspects of my life.		0.91		0.56
Correlation between the two factors	0.52 ***		0.60 ***	

Note: MBI = muscle-oriented body image; MB = muscle-oriented behavior. *** *p* < 0.001.

**Table 3:** Standardized factor loadings and factor correlations for the Muscle Dysmorphic Disorder Inventory.

Items	Standardized Factor Loadings					
	University Sample			General Adult Sample		
	DFS	AI	FI	DFS	AI	FI
1. I think my body is too small.	0.77			0.70		
4. I wish I could get bigger.	0.78			0.75		
5. I think my chest is too small.	0.84			0.77		
6. I think my legs are too thin.	0.85			0.67		
8. I wish my arms were bigger.	0.72			0.69		
2. I wear loose clothing so that people can’t see my body.		0.75			0.68	
3. I hate my body.		0.83			0.62	
7. I feel like I have too much body fat.		0.76			0.55	
9. I am very shy about letting people see me with my shirt off.		0.85			0.70	
10. I feel anxious when I miss one or more workout days.			0.91			0.80
11. I pass up social activities with friends because of my workout schedule.			0.89			0.78
12. I feel depressed when I miss one or more workout days.			0.91			0.85
13. I pass up chances to meet new people because of my workout schedule.			0.87			0.76
Correlation among the three factors	1	2	3	1	2	3
1. Drive for size	-			-		
2. Appearance intolerance	0.59 ***	-		0.37 ***	-	
3. Functional impairment	0.62 ***	0.76 ***	-	0.47 ***	0.52 ***	-

Note: DFS = drive for size; AI = appearance intolerance; FI = functional impairment. *** *p* < 0.001.

**Table 4:** Measurement invariance tests by samples for the DMS and MDDI.

	$\chi^{2}$	*df*	CFI	TLI	SRMR	ΔCFI	ΔSRMR
DMS							
Configural Model	939.943 ***	172	0.955	0.946	0.088		
Metric Model	1061.090 ***	185	0.949	0.942	0.098	−0.006	0.010
Scalar Model	1127.526 ***	243	0.949	0.956	0.089	0.000	−0.009
MDDI							
Configural Model	489.644 ***	122	0.958	0.946	0.071		
Metric Model	471.001 ***	132	0.961	0.954	0.074	0.008	0.003
Scalar Model	584.664 ***	168	0.952	0.955	0.072	−0.009	−0.002

Notes: DMS = Drive for Muscularity Scale; MDDI = Muscle Dysmorphic Disorder Inventory. *** *p* < 0.001.

**Table 5:** Correlations among the variables included in the current study.

	1	2	3	4	5	6	7	8	9	10	11	12	13	14
1. MOET	-	0.47 ***	0.26 ***	0.57 ***	0.53 ***	0.26 ***	0.45 ***	0.62 ***	0.38 ***	0.39 ***	0.62 ***	0.24 ***	0.16 **	0.01
2. DMS	0.70 ***	-	0.88 ***	0.80 ***	0.64 ***	0.56 ***	0.48 ***	0.52 ***	0.37 ***	0.38 ***	0.45 ***	0.17 **	0.04	0.02
3. DMS-MBI	0.43 ***	0.86 ***	-	0.43 ***	0.53 ***	0.52 ***	0.42 ***	0.34 ***	0.24 ***	0.28 ***	0.27 ***	0.10	−0.04	−0.002
4. DMS-MB	0.77 ***	0.88 ***	0.51 ***	-	0.56 ***	0.42 ***	0.40 ***	0.56 ***	0.40 ***	0.38 ***	0.52 ***	0.20 ***	0.13 *	0.03
5. MDDI	0.58 ***	0.66 ***	0.61 ***	0.55 ***	-	0.78 ***	0.84 ***	0.80 ***	0.45 ***	0.51 ***	0.57 ***	0.38 ***	0.10	0
6. MDDI-DFS	0.32 ***	0.57 ***	0.62 ***	0.37 ***	0.76 ***	-	0.42 ***	0.45 ***	0.28 ***	0.37 ***	0.21 ***	0.001	−0.24 ***	−0.02
7. MDDI-AI	0.32 ***	0.29 ***	0.28 ***	0.23 ***	0.69 ***	0.24 ***	-	0.58 ***	0.41 ***	0.44 ***	0.62 ***	0.61 ***	0.34 ***	−0.04
8. MDDI-FI	0.66 ***	0.61 ***	0.43 ***	0.62 ***	0.78 ***	0.39 ***	0.39 ***	-	0.38 ***	0.45 ***	0.58 ***	0.27 ***	0.18 **	0.09
9. CIA	0.54 ***	0.38 ***	0.26 ***	0.39 ***	0.57 ***	0.27 ***	0.54 ***	0.50 ***	-	0.62 ***	0.42 ***	0.26 ***	0.16 **	0.06
10. K6	0.34 ***	0.24 ***	0.19 ***	0.23 ***	0.45 ***	0.27 ***	0.41 ***	0.35 ***	0.66 ***	-	0.46 ***	0.28 ***	0.09	0.05
11. EDE-QS	0.69 ***	0.50 ***	0.27 ***	0.59 ***	0.52 ***	0.19 ***	0.49 ***	0.52 ***	0.58 ***	0.39 ***	-	0.50 ***	0.39 ***	0.004
12. EDI-BD	0.14 **	0.12 *	0.14 **	0.07	0.37 ***	0.004	0.68 ***	0.22 ***	0.45 ***	0.33 ***	0.43 ***	-	0.47 ***	0.03
13. BMI	0.06	−0.07	−0.13 **	−0.01	−0.004	−0.31 ***	0.29 ***	0.07	0.12 *	−0.02	0.28 ***	0.47 ***	-	0.10
14. Age	−0.05	−0.10 *	−0.09	−0.08	−0.02	−0.07	−0.06	0.08	−0.10	−0.12 *	−0.05	0.05	0.33 ***	-
Students *M*	8.93	2.15	2.62	1.73	22.09	8.24	7.82	6.07	7.33	10.67	6.24	31.40	22.13	18.92
*SD*	8.57	0.86	1.21	0.84	8.03	3.60	3.74	2.59	7.28	3.85	6.84	10.47	3.92	1.04
General adults *M*	22.18	3.18	3.65	2.70	32.45	12.98	9.51	9.96	9.08	5.43	10.20	28.18	22.28	28.53
*SD*	10.56	0.79	0.93	0.89	8.16	4.04	3.31	3.56	7.08	3.49	6.57	10.00	2.80	5.56

Notes: University students’ correlations are on the top diagonals, and general adults’ correlations are on the bottom diagonals. MOET = Muscularity-Oriented Eating Test; DMS = drive for muscularity scale; MBI-DMS = muscle-oriented body image; MB-DMS = muscle-oriented behavior; MDDI = Muscle Dysmorphic Disorder Inventory; MDDI-DS = drive for size subscale of the Muscle Dysmorphic Disorder Inventory; AI = appearance intolerance subscale of the Muscle Dysmorphic Disorder Inventory; FI = functional impairment subscale of the Muscle Dysmorphic Disorder Inventory; CIA = Clinical Impairment Assessment 3.0; K6 = Kessler Psychological Distress Scale; EDE-QS = Eating Disorder Examination Questionnaire Short; EDI-BD = body dissatisfaction subscale of the Eating Disorder Inventory. * *p* < 0.05. ** *p* < 0.01. *** *p* < 0.001.

**Table 6:** Incremental contributions of the Muscularity-Oriented Eating Test (MOET), the drive for muscularity scale (DMS) and the Muscle Dysmorphic Disorder Inventory (MDDI) scores to the relevant criterion variables.

	**University Sample**				**General Adult Sample**			
	*F*	Total *R*^2^	Δ *R* ^2^	β	*F*	Total *R*^2^	Δ *R* ^2^	β
Criterion: psychosocial impairment (CIA)								
Step 1	15.07 ***	0.19 ***	-		68.82 ***	0.40 ***	-	
Age				0.04				−0.04
BMI				−0.04				−0.15 **
Thinness-oriented body dissatisfaction (EDI-BD)				0.09				0.31 ***
Traditional eating disorder symptomatology (EDE-QS)				0.40 ***				0.49 ***
Step 2	13.81 ***	0.21 ***	0.02 **		69.10 ***	0.46 ***	0.06 ***	
Age				0.04				−0.05
BMI				−0.02				−0.12 *
Thinness-oriented body dissatisfaction				0.11				0.36 ***
Traditional eating disorder symptomatology				0.27 ***				0.22 ***
MOET scores				0.19 **				0.34 ***
Criterion: muscularity-oriented disordered eating (MOET)								
Step 1	42.78 ***	0.40 ***	-		108.73 ***	0.52 ***	-	
Age				0.03				0.02
BMI				−0.10				−0.10 *
Thinness-oriented body dissatisfaction				−0.08				−0.16 ***
Traditional eating disorder symptomatology				0.71 ***				0.79 ***
Step 2	39.81 ***	0.43 ***	0.04 ***		158.43 ***	0.66 ***	0.14 ***	
Age				0.02				0.02
BMI				−0.07				0.01
Thinness-oriented body dissatisfaction				−0.08				−0.14 ***
Traditional eating disorder symptomatology				0.60 ***				0.52 ***
DMS scores				0.22 ***				0.46 ***
Criterion: muscle dysmorphic disorder symptoms (MDDI)								
Step 1	34.44 ***	0.34 ***	-		56.03 ***	0.35 ***	-	
Age				0.03				0.09
BMI				−0.19 ***				−0.31 ***
Thinness-oriented body dissatisfaction				0.20 ***				0.31 ***
Traditional eating disorder symptomatology				0.53 ***				0.47 ***
Step 2	63.14 ***	0.55 ***	0.21 ***		102.50 ***	0.56 ***	0.21 ***	
Age				−0.002				0.08
BMI				−0.13 **				−0.19 ***
Thinness-oriented body dissatisfaction				0.21 ***				0.32 ***
Traditional eating disorder symptomatology				0.26 ***				0.16 ***
DMS scores				0.51 ***				0.54 ***
Criterion: psychosocial impairment								
Step 1	17.25 ***	0.21 ***	-		68.82 ***	0.40 ***	-	
Age				0.03				−0.04
BMI				−0.04				−0.15 **
Thinness-oriented body dissatisfaction				0.07				0.31 ***
Traditional eating disorder symptomatology				0.44 ***				0.49 ***
Step 2	20.02 ***	0.28 ***	0.07 ***		70.55 ***	0.46 ***	0.06 ***	
Age				0.02				−0.07
BMI				0.03				−0.05
Thinness-oriented body dissatisfaction				0.001				0.21 ***
Traditional eating disorder symptomatology				0.27 ***				0.34 ***
MDDI scores				0.33 ***				0.31 ***

Notes: MOET = Muscularity-Oriented Eating Test; DMS = drive for muscularity scale; MDDI = Muscle Dysmorphic Disorder Inventory; CIA = Clinical Impairment Assessment 3.0; EDE-QS = Eating Disorder Examination Questionnaire Short; EDI-BD = body dissatisfaction subscale of the Eating Disorder Inventory. * *p* < 0.05. ** *p* < 0.05. *** *p* < 0.001.

## Data Availability

The dataset used during the current study is available from the corresponding author upon reasonable request.
